# Reduced 24-h Sodium Excretion Is Associated With a Disturbed Plasma Acylcarnitine Profile in Vasovagal Syncope Children: A Pilot Study

**DOI:** 10.3389/fped.2020.00098

**Published:** 2020-03-11

**Authors:** Jinqing Song, Chunyan Tao, Guozhen Chen, Selena Chen, Wenrui Xu, Junbao Du, Yanling Yang, Yaqian Huang

**Affiliations:** ^1^Department of Pediatrics, Peking University First Hospital, Beijing, China; ^2^Department of Pediatrics, The Affiliated Yantai Yuhuangding Hospital of Qingdao University, Yantai, China; ^3^Division of Biological Sciences, University of California, San Diego, La Jolla, CA, United States

**Keywords:** carnitine, acylcarnitine, low sodium intake, vasovagal syncope, children

## Abstract

**Objective:** To investigate if the low sodium intake is associated with the plasma carnitine and acylcarnitine profile in children with vasovagal syncope (VVS).

**Materials and Methods:** Twenty-six children suffering from VVS were recruited in the present study and divided into a group of low urinary sodium excretion or a group of normal urinary sodium excretion according to the excretion of 24-h urinary sodium <3 or 3–6 g, respectively. The excretion of 24-h urinary sodium was detected with ion-selective electrode approach. Plasma carnitine and acylcarnitine concentrations were measured with tandem mass spectrometry. Each participant completed the head-up tilt test. The demographics, clinical characteristics, hemodynamic parameters and plasma carnitine and acylcarnitine concentrations were compared between the two groups. A bivariate correlation between plasma acylcarnitine profiles and the excretion of 24-h urinary sodium was conducted with Spearman's correlation coefficients.

**Results:** Of the enrolled VVS patients, 14 patients were assigned to the group of low urinary sodium excretion and the remaining 12 patients were assigned to the group of normal urinary sodium excretion. Symptoms of fatigue were more prevalent in the group of low urinary sodium excretion than in the group of normal urinary sodium excretion (*p* = 0.009). Aside from fatigue, no other differences in the demographics, clinical characteristics or hemodynamic parameters during the head-up tilt test were found between the two groups (*p* > 0.05). Concentrations of plasma tiglylcarnitine (C5:1), hydroxyhexadecanoylcarnitine (C16OH), hydroxyoctadecanoylcarnitine (C18OH), and carnitine C22 were significantly higher in the group of low urinary sodium excretion than in the group of normal urinary sodium excretion (all *p-*values = 0.048); moreover, they were all negatively correlated with 24-h urinary sodium levels (all *p*-values = 0.016). There were no differences between the two groups in other acylcarnitines or free carnitine.

**Conclusions:** Reduced excretion of 24-h urinary sodium is associated with a disturbed plasma acylcarnitine profile in children with VVS. The findings suggest that restricted sodium intake-induced disturbance of plasma acylcarnitines and related cellular energy metabolism might be involved in the pathogenesis of VVS in children.

## Introduction

Vasovagal syncope (VVS) is the main form of syncope in children and adolescents and is characterized by syncopal attack and hemodynamic abnormalities during the head-up tilt test (HUTT), including systemic arterial hypotension, bradycardia, or both. It has been reported that VVS accounts for more than 60% of all pediatric syncopal cases (1, 2). Approximately 30% of affected individuals suffer from recurrent syncopal episodes, which significantly impair their quality of life (3, 4). Under such circumstances, efficient interventions are required. However, the exact underlying mechanisms of VVS are not fully understood, which results in an unsatisfactory outcome of treatment to some extent (5, 6). Therefore, elucidation of the complex mechanisms of VVS is particularly important. Fatigue, which is related to energy metabolic dysfunction, is common in patients with VVS (7). Moreover, it has been established that skeletal muscle extensively accumulates carnitine and acylcarnitines, which are pivotal in cellular energy production because of their role in transporting long-chain fatty acids from the cellular matrix into the matrix of mitochondrion for subsequent beta-oxidation (8, 9). Nevertheless, it is unclear whether a disturbance of carnitine and acylcarnitine profiles is associated with the pathogenesis of VVS. Low sodium intake is one proposed mechanism of VVS and leads to hypovolemia and alterations in biologically active substances which are likely to contribute to syncopal occurrence, including renin, aldosterone, and catecholamine (10–12). As a result, supplementation of fluid and sodium is a rationally efficient method to treat VVS (13). A possible relationship between the metabolism of carnitine and acylcarnitines and the reducing intake of sodium was mentioned among adults with hypertension (14). However, it is unclear whether low sodium intake in patients with VVS leads to abnormal changes in carnitine and acylcarnitines, which mediates the occurrence of syncope.

Therefore, the present research was undertaken to explore if the low sodium intake is associated with the plasma carnitine and acylcarnitine profile in pediatric VVS patients and discuss the possible underlying mechanisms.

## Materials and Methods

### Study Subjects

Twenty-six patients (19 girls and 7 boys, aged 12.5 ± 2.7 years old) with VVS were enrolled in the present research. They visited the Child Syncope Center at Peking University First Hospital from August 2018 to September 2019. All patients underwent an excretion test of 24-h urinary sodium. Based on the excretion levels of 24-h urinary sodium, patients were divided into the group of low urinary sodium excretion (excretion level <3 g) or the group of normal urinary sodium excretion (excretion level between 3 and 6 g) (2). No patients were overweight or obese, nor had associated disorders of diabetes mellitus, thyroid dysfunction, renal dysfunction, hepatic dysfunction, or cardiac dysfunction or symptoms and signs of fever, vomiting, diarrhea or dehydration. The criteria for the diagnosis of pediatric VVS were as follows: (1) syncopal events that were often induced by predisposing factors including rapid postural alterations, long-term standing, and emotional stimulation, etc.; (2) a positive response to the HUTT; as well as (3) excluding other syncopal disorders (15, 16). The Ethics Committee of Peking University First Hospital approved this study. We conducted the study in line with the ethical criteria in the Declaration of Helsinki. The legal guardians of each patient signed the informed consent.

### Protocol for the HUTT

All enrolled patients underwent strict inspections to exclude cardiogenic, neurological, and metabolic disorders. Patients then completed the HUTT. This test was conducted in a dimly lit, warm and quiet room between 8:30 and 12:00 a.m. Patients were required to fast and the medication impacting autonomic nervous function was avoided at least 5 half-life times before the HUTT. They were placed on an autonomic stretcher (HUT-821; Beijing Juchi, Beijing, China) for 10–20 min to maintain a stable hemodynamic condition. Then, they were positioned at 60° passively for as long as 45 min or until a positive response occurred. A positive response to the HUTT was according to the criteria described previously (15, 16).

### Protocol for the 24-h Urinary Sodium Excretion Test

Each patient as well as his/her caretakers were introduced to a standard urine collection process in advance. Girls were required to make a collection of 24-h urine samples during non-menstrual periods. The sodium-selective electrode approach (Cobas 6000, Roche, Basel, Switzerland) was done for measuring the excretion of 24-h urinary sodium. The formula is as follows (17): excretion of 24-h urinary sodium = concentration of sodium × total 24-h urine volume.

### Measurement of Plasma Carnitine and Acylcarnitines

All VVS patients fasted for at least 4 h before blood sampling at ~8:00 a.m. Two milliliters of blood were drawn by venipuncture and collected in an ethylenediaminetetraacetic acid anticoagulant tube. After being centrifugated at 2,000 g for 20 min, plasma was extracted at 4°C and then preserved at −80°C until tested. The collected plasma was thawed at one time for the measurement of the carnitine and acylcarnitine profile on a tandem mass spectrometer (MS/MS, API3200, Applied Biosystems, California, USA). The concentrations of carnitine and acylcarnitines were calculated automatically using Chemo View software (NeoLynx, Waters, Massachusetts, USA). All the testing work was done by a professionally trained investigator.

### Statistical Analysis

The data were processed with SPSS software 22.0 (IBM Corp., NY, USA). Continuous variables are stated as the mean ± SD. And between two groups, the differences were measured by Student's *t*-test or Mann-Whitney *U*-test according to the Shapiro-Wilk test consequences. Categorical variables are presented as numbers, and Fisher's exact test was applied for comparison. A bivariate correlation between data with non-normal distribution was conducted with Spearman's correlation coefficients. Significance was considered when *p*-value <0.05.

## Results

### Basic Information of VVS Patients

Among the enrolled VVS patients, the group of low urinary sodium excretion included 14 patients with a 24-h urinary sodium excretion of 0.8–2.7 g (mean 2.1 ± 0.6 g), and the group of normal urinary sodium excretion included 12 children with a 24-h urinary sodium excretion of 3.1–5.9 g (mean 3.8 ± 0.8 g). No differences existed in the demographics, and clinical and hemodynamic characteristics between the two groups (*p* > 0.05, Table 1). However, more children in the group of low urinary sodium excretion reported fatigue than in the group of normal urinary sodium excretion (*p* = 0.009, Table 1).

**Table 1:** Baseline characteristics and hemodynamics in the head-up tilt test in vasovagal syncope patients.

**Items**	**Low urinary sodium excretion group (*n* = 14)**	**Normal urinary sodium excretion group (*n* = 12)**	***t/Z/χ*^**2**^ value**	***p*-value**
Gender (*n*, M/F)	3/11	4/8	—^†^	0.665
Age (years)	12.1 ± 3.0	13.0 ± 2.4	−0.788	0.439
Body mass index (kg/m^2^)	17.6 ± 2.3	20.0 ± 3.9	−1.916	0.067
Symptom duration (months)	29.6 ± 37.1^*^	17.1 ± 19.5^*^	−0.646	0.518
Numbers of syncope (times)	4 ± 3^*^	4 ± 2^*^	−1.157	0.247
Duration of unconsciousness (min)	5.2 ± 8.3^*^	1.5 ± 1.7^*^	−1.066	0.286
Fatigue (*n*, yes/no)	13/1	5/7	—^†^	0.009
Supine HR (beats/min)	77 ± 14^*^	77 ± 14	−0.180	0.857
Supine SBP (mmHg)	106 ± 9	110 ± 11^*^	−1.185	0.236
Supine DBP (mmHg)	62 ± 6	66 ± 9	−1.070	0.295
Time to positive response in HUTT (min)	31 ± 11^*^	26 ± 13	−1.159	0.246
Positive HR in HUTT (beats/min)	116 ± 32	114 ± 16	0.270	0.789
Positive SBP in HUTT (mmHg)	72 ± 5	71 ± 5	0.567	0.576
Positive DBP in HUTT (mmHg)	48 ± 7^*^	48 ± 4	−0.438	0.661

*HR, heart rate; SBP, systolic blood pressure; DBP, diastolic blood pressure; HUTT, head-up tilt test; Data are mean ± SD or numbers*.

* *Non-normality*.

† *Fisher's exact test*.

### Plasma Carnitine and Acylcarnitine Profile in VVS Patients

Levels of several acylcarnitines, namely tiglylcarnitine (C5:1) (*p* = 0.048), hydroxyhexadecanoylcarnitine (C16OH) (*p* = 0.048), hydroxyoctadecanoylcarnitine (C18OH) (*p* = 0.048), and carnitine C22 (*p* = 0.048), were higher in the group of low urinary sodium excretion than in the group of normal urinary sodium excretion (Table 2). No statistically significant differences were showed between the two groups in other acylcarnitines or free carnitine (C2, *p* = 0.504; C3, *p* = 0.777; C3DC, *p* = 0.803; C4, *p* = 0.620; C5, *p* = 1.000; C5DC, *p* = 0.912; C5OH, *p* = 0.654; C6, *p* = 0.312; C6DC, *p* = 0.354; C8, *p* = 0.288; C8:1, *p* = 0.313; C10, *p* = 0.359; C10:1, *p* = 0.086; C10:2, *p* = 0.192; C12, *p* = 0.720; C12:1, *p* = 0.083; C14, *p* = 0.845; C14:1, *p* = 0.565; C14:2, *p* = 0.146; C14DC, *p* = 0.181; C14OH, *p* = 0.234; C16, *p* = 0.897; C16:1, *p* = 0.863; C16:1OH, *p* = 0.725; C18, *p* = 0.958; C18:1, *p* = 0.813; C18:1OH, *p* = 0.481; C18:2, *p* = 0.813; C20, *p* = 0.105; C24, *p* = 0.457; C26, *p* = 0.355; C5/C2, *p* = 0.297; C2/C0, *p* = 0.366; C3/C2, *p* = 0.602; C3/C0, *p* = 1.000; C8/C2, *p* = 0.867; C14:1/C16, *p* = 0.909; free carnitine, *p* = 0.897, Table 2).

**Table 2:** Plasma carnitine and acylcarnitines in vasovagal syncope patients.

**Items**	**Low urinary sodium excretion group (*n* = 14)**	**Normal urinary sodium excretion group (*n* = 12)**	***t/Z* value**	***p-*value**
Free carnitine	23.657 ± 5.937	23.388 ± 4.33	0.130	0.897
C2	3.110 ± 1.237	2.885 ± 1.005^*^	−0.669	0.504
C3	0.204 ± 0.066	0.206 ± 0.071^*^	−0.284	0.777
C3DC	0.019 ± 0.010^*^	0.018 ± 0.006^*^	−0.249	0.803
C4	0.101 ± 0.039	0.108 ± 0.038	−0.502	0.620
C5	0.045 ± 0.015	0.045 ± 0.016	0.000	1.000
C5:1	0.013 ± 0.005^*^	0.010 ± 0.000	−1.974	0.048
C5DC	0.054 ± 0.023^*^	0.047 ± 0.010^*^	−0.110	0.912
C5OH	0.029 ± 0.010^*^	0.027 ± 0.005^*^	−0.448	0.654
C6	0.032 ± 0.012	0.029 ± 0.012^*^	−1.012	0.312
C6DC	0.052 ± 0.022^*^	0.041 ± 0.007^*^	−0.926	0.354
C8	0.068 ± 0.026	0.061 ± 0.032^*^	−1.063	0.288
C8:1	0.125 ± 0.035	0.122 ± 0.064^*^	−1.009	0.313
C10	0.086 ± 0.033	0.073 ± 0.035	0.935	0.359
C10:1	0.102 ± 0.023	0.092 ± 0.034^*^	−1.715	0.086
C10:2	0.021 ± 0.006^*^	0.018 ± 0.006^*^	−1.305	0.192
C12	0.029 ± 0.012	0.028 ± 0.015^*^	−0.359	0.720
C12:1	0.052 ± 0.015^*^	0.043 ± 0.014^*^	−1.732	0.083
C14	0.019 ± 0.010^*^	0.018 ± 0.012^*^	−0.195	0.845
C14:1	0.047 ± 0.013	0.045 ± 0.012^*^	−0.575	0.565
C14:2	0.019 ± 0.007^*^	0.016 ± 0.009^*^	−1.452	0.146
C14DC	0.011 ± 0.004^*^	0.010 ± 0.000	−1.336	0.181
C14OH	0.006 ± 0.005^*^	0.003 ± 0.005^*^	−1.190	0.234
C16	0.114 ± 0.118^*^	0.110 ± 0.095^*^	−0.130	0.897
C16:1	0.016 ± 0.012^*^	0.015 ± 0.008^*^	−0.173	0.863
C16:1OH	0.006 ± 0.008^*^	0.004 ± 0.005^*^	−0.352	0.725
C16OH	0.003 ± 0.005^*^	0.000 ± 0.000	−1.974	0.048
C18	0.036 ± 0.032^*^	0.038 ± 0.037^*^	−0.053	0.958
C18:1	0.118 ± 0.125^*^	0.101 ± 0.100^*^	−0.237	0.813
C18:1OH	0.003 ± 0.005^*^	0.002 ± 0.004^*^	−0.704	0.481
C18:2	0.076 ± 0.073^*^	0.063 ± 0.056^*^	−0.236	0.813
C18OH	0.003 ± 0.005^*^	0.000 ± 0.000	−1.974	0.048
C20	0.004 ± 0.005^*^	0.001 ± 0.003^*^	−1.620	0.105
C22	0.003 ± 0.005^*^	0.000 ± 0.000	−1.974	0.048
C24	0.009 ± 0.003^*^	0.008 ± 0.004^*^	−0.743	0.457
C26	0.009 ± 0.003^*^	0.010 ± 0.000	−0.926	0.355
C5/C2	0.014 ± 0.006^*^	0.017 ± 0.007^*^	−1.043	0.297
C2/C0	0.121 ± 0.043^*^	0.108 ± 0.029^*^	−0.905	0.366
C3/C2	0.069 ± 0.027^*^	0.075 ± 0.028	−0.522	0.602
C3/C0	0.010 ± 0.000	0.010 ± 0.000	0.000	1.000
C8/C2	0.021 ± 0.009	0.022 ± 0.006^*^	−0.167	0.867
C14:1/C16	0.736 ± 0.373	0.717 ± 0.465	0.116	0.909

*Data are mean ± SD*.

* *Non-normality*.

### Correlations Between the Excretion of 24-h Urinary Sodium and Plasma Acylcarnitines

Further examining the association between the excretion levels of 24-h urinary sodium and the abovementioned plasma acylcarnitines with significant difference, negative Spearman's correlation coefficients were found (C5:1, *r* = −0.469, *p* = 0.016; C16OH, *r* = −0.469, *p* = 0.016; C18OH, *r* = −0.469, *p* = 0.016; and C22, *r* = −0.469, *p* = 0.016) in all study patients.

## Discussion

In mitochondria, carnitine and acylcarnitines are a series of essential molecules that play an obligatory part in the long-chain fatty acid beta-oxidation. Analyzing carnitine and acylcarnitine profile may help in the investigation and elucidation of metabolic derangements. The acylcarnitine profile displays a characteristic alteration in inherited metabolic diseases, such as short chain and medium chain acyl coenzyme A dehydrogenase deficiency, and carnitine acylcarnitine translocase deficiency (18). Moreover, it was reported that changes in the acylcarnitine profile were related to the development of IgA nephropathy and diabetes mellitus (19, 20). In the study, we observed that in VVS patients, some plasma acylcarnitines (C5:1, C16OH, C18OH, C22) were higher in the group of low urinary sodium excretion than in the group of normal urinary sodium excretion. Moreover, the excretion of 24-h urinary sodium was negatively correlated with these plasma acylcarnitines. Patients suffering from VVS with low urinary sodium excretion were more susceptible to experiencing symptoms of fatigue than those in the group of normal urinary sodium excretion.

The mechanisms by which low sodium loading results in a disturbed acylcarnitine profile have not been explored. Derkach et al. also found a phenomenon in a DASH-sodium trial among participants with high blood pressure, that plasma metabolites of acylcarnitines (butyrylcarnitine and valerylcarnitine) were increased with sodium reduction (14). On the other hand, some studies showed a significant increase in renin-angiotensin-aldosterone system activity with low sodium intake (12). The activated renin-angiotensin-aldosterone system is capable of inducing oxidative stress (21, 22), which could diminish the activity of enzymes in metabolic processes. Therefore, we inferred that low sodium loading might impair the activity of enzymes involved in the long-chain fatty acid beta-oxidation. The inefficient long-chain fatty acid beta-oxidation could promote the accumulation of acylcarnitine byproducts from substrate catabolism, which are capable of passing across the mitochondrial membrane and the cell membrane (19, 23, 24). More convincingly, 24-h urinary sodium excretion was found to be negatively correlated with the concentration of several acylcarnitines in our study, which suggested that reduced dietary sodium intake might be associated with abnormal fatty acid metabolism.

As a high-energy-consuming organ, skeletal muscle needs fatty acids to supplement essential energy during exercise or stress (25). Insufficient energy leads to fatigue. Large numbers of VVS patients complain of fatigue before or after syncope occurrence (7). In our Syncope Unit, we also observed several patients experiencing fatigue for long durations of time. In the present study, patients with low urinary sodium excretion were more prone to experience fatigue than those with normal urinary sodium excretion. This highlights that some VVS patients are disturbed by energy metabolism dysfunction, which is likely induced, at least in part, by low sodium intake.

The excretion of 24-h urinary sodium, a standard criterion for the measurement of sodium intake (26), is applied in many investigations to explore the relationship between sodium intake and development of diverse disorders. Kieneker et al. conducted a study in 7,330 individuals and found a strong relationship between low urinary sodium level and an increased stroke risk (27). Moreover, many investigations have showed that abnormal sodium intake is related to increased mortality and cardiovascular events (28, 29). The relationship between sodium intake and outcomes of health should be a U-shape, where high and low sodium intake indicated a risk factor for negative outcomes. We suggested that low sodium intake might decrease the long-chain fatty acid beta-oxidation rate and increase the plasma concentration of acylcarnitines in VVS patients. Additional and longer investigations should be performed to determine whether low sodium intake can predict the outcomes of affected individuals.

In summary, increased plasma concentration of acylcarnitines in VVS patients with low urinary sodium excretion was reported for the first time. In addition to VVS, postural tachycardia syndrome is another subtype of orthostatic intolerance with fatigue, palpitation and dizziness as well as syncope in the childhood (30, 31). Furthermore, some postural tachycardia syndrome patients are also characterized with low sodium intake (32, 33). As such, we speculate that the restricted sodium intake- induced disturbance of plasma acylcarnitines and cellular energy metabolism might be associated with the pathogenesis of not only VVS, but also other subtypes of orthostatic intolerance in children, which merits further investigations. Also, there were some limitations, such as a small sample size, the single-center observational study design and the lack of healthy control participants. Future multicenter-based, larger sample-sized and mechanistic studies should be performed to explore the significance of low-sodium-intake-associated energy metabolism in pathogenesis of VVS.

## Data Availability Statement

The datasets generated for this study are available on request to the corresponding author.

## Ethics Statement

The studies involving human participants were reviewed and approved by the Ethics Committee of Peking University First Hospital. Written informed consent to participate in this study was provided by the participants' legal guardian/next of kin.

## Author Contributions

JS had the primary responsibility for the protocol development, examined the plasma concentrations of carnitine and acylcarnitines. CT was responsible for patient enrollment, data collection, and preliminary data analysis. JS and CT wrote the drafted manuscript. GC, SC, JD, and WX assisted with the data collection and data analysis. GC, JD, and SC revised the manuscript and gave important advice on the subject. WX, YY, and YH supervised the design and execution of this study, checked data analysis, revised the manuscript, and had a final approval of the submitted manuscript. All authors have read and approved the final manuscript and assumed full responsibility for its contents.

### Conflict of Interest

The authors declare that the research was conducted in the absence of any commercial or financial relationships that could be construed as a potential conflict of interest.

## Abbreviations

VVS: vasovagal syncope
HUTT: head-up tilt test.
